# Aldehyde Dehydrogenase 2 Has Cardioprotective Effects on Myocardial Ischaemia/Reperfusion Injury via Suppressing Mitophagy

**DOI:** 10.3389/fphar.2016.00101

**Published:** 2016-04-21

**Authors:** Wenqing Ji, Shujian Wei, Panpan Hao, Junhui Xing, Qiuhuan Yuan, Jiali Wang, Feng Xu, Yuguo Chen

**Affiliations:** ^1^Department of Emergency, Qilu Hospital, Shandong UniversityJinan, China; ^2^Chest Pain Center, Qilu Hospital, Shandong UniversityJinan, China; ^3^Institute of Emergency and Critical Care Medicine, Shandong UniversityJinan, China; ^4^Key Laboratory of Emergency and Critical Care Medicine of Shandong Province, Qilu Hospital, Shandong UniversityJinan, China; ^5^Key Laboratory of Cardiovascular Remodeling and Function Research, Chinese Ministry of Education and Chinese Ministry of Public Health, Qilu Hospital, Shandong UniversityJinan, China

**Keywords:** ALDH2, Alda-1, myocardial ischemia/reperfusion, mitophagy, PINK1/Parkin, reactive oxygen species, mitochondrial superoxide

## Abstract

Mitophagy, a selective form of autophagy, is excessively activated in myocardial ischemia/reperfusion (I/R). The study investigated whether aldehyde dehydrogenase 2 (ALDH2) exerted its cardioprotective effect by regulating mitophagy. Myocardial infarct size and apoptosis after I/R in rats were ameliorated by Alda-1, an ALDH2 activator, and aggravated by ALDH2 inhibition. Both in I/R rats and hypoxia/reoxygenation H9C2 cells, ALDH2 activation suppressed phosphatase and tensin homolog-induced putative kinase 1 (PINK1)/Parkin expression, regulating mitophagy, by preventing 4-hydroxynonenal, reactive oxygen species and mitochondrial superoxide accumulation. Furthermore, the effect was enhanced by ALDH2 inhibition. Thus, ALDH2 may protect hearts against I/R injury by suppressing PINK1/Parkin–dependent mitophagy.

## Introduction

Mitochondrial aldehyde dehydrogenase 2 (ALDH2) is an allosteric tetrameric enzyme responsible for the metabolism or detoxification of acetaldehyde and other toxic aldehydes, in particular 4-hydroxynonenal (4HNE), as well as mitochondrial oxidative ATP generation and reactive oxygen species (ROS) generation (Li et al., 2006; Ren, 2007; Li and Ren, 2008). ALDH2 activity can affect the cellular response to oxidative stress both *in vitro* and *in vivo* (Li et al., 2004; Wenzel et al., 2008). It can be affected by gene regulation, post-translational modifications and pharmacological intervention (Chen et al., 2008; Choi et al., 2011; Lu et al., 2011). ALDH2 can be directly activated by the activator Alda-1 and inhibited by the inhibitor Daidzin.

A recent study revealed an important role of ALDH2 in cardioprotection against ischemia injury (Chen et al., 2008). ALDH2 has a dual regulatory paradox in cardioprotecting against ischemia/reperfusion (I/R) injury via autophagy (Ma et al., 2011). Autophagy is triggered by energy depletion, oxidative stresses, protein aggregates and damaged organelles (Tannous et al., 2008; Kim et al., 2013). Despite many studies demonstrating the benefits of autophagy, excessive autophagy can induce cell death in myocardial I/R injury (Matsui et al., 2007; Qiao et al., 2013).

Mitophagy, a selective form of autophagy, is a specific process for degradation of dysfunctional or damaged mitochondria to maintain a healthy mitochondria population and mitochondrial quality (Narendra et al., 2008; Poole et al., 2008). Starving, hypoxemia, and ROS may trigger mitophagy, found associated with several forms of neurodegeneration and cardiovascular diseases (Youle and Narendra, 2011; Dorn and Kitsis, 2015). Mitophagy can be regulated by the phosphatase and tensin homolog-induced putative kinase 1 (PINK1)/Parkin pathway (Springer and Kahle, 2011). Upon mitochondrial damage, PINK1, a mitochondria-localized serine/threonine kinase, globally accumulates on depolarized mitochondria and recruits Parkin from the cytosol to mitochondria. Parkin, an E3 ubiquitin ligase, catalyzes the polyubiquitination of several substrates and triggers whole-mitochondrial engulfment by autophagosomes and subsequent degradation via the autophagosome lysosome pathway (Geisler et al., 2010).

Nevertheless, the precise role of mitophagy in myocardial I/R injury is unclear. Here, we explored the potential involvement and function of mitophagy in the ALDH2-elicited cardioprotective effect in myocardial I/R injury *in vivo* and *in vitro*. We first confirmed that ALDH2 is cardioprotective in myocardial I/R injury in rats, then showed that ALDH2 inhibited excessive mitophagy and increased the survival of I/R cardiomyocytes by reducing 4HNE and ROS levels. Finally, we investigated the potential mechanism of ALDH2 in protecting against myocardial I/R injury.

## Materials and Methods

### Animals

All animal procedures were performed in accordance with the guidelines of the Institute Animal User and Ethical Committees at Shandong University were approved by it. Wistar rats were purchased from Vital River Laboratory Animal Technology Co. (Beijing) and were housed at a temperature-controlled room under a 12:12 h light-dark circadian cycle. Six-to-eight-week old adult male rats weighing 200–250 g were randomly assigned to 4 groups (*n* = 12/group) for treatment:SHAM, I/R, I/R plus Alda-1 (I/R + Alda-1), and I/R plus Daidzin (I/R + Daidzin). Alda-1 and Daidzin were dissolved in DMSO. Rats were administered 10 mg/kg Alda-1 (SML0462, Sigma, USA) or 100 mg/Kg Daidzin (SML30408, Sigma, USA) through intramyocardial injection into the left ventricular myocardium 5 min before 30 min ischemia followed by 120 min reperfusion. Rats were anesthetized with pentobarbital sodium (100 mg/kg body weight, intraperitoneal injection), intubated and ventilated with oxygen (Rodent Ventilator, Harvard Apparatus, Millis, MA, USA). The core temperature was maintained at 37°C. For I/R rats, we occluded the left anterior descending artery (LAD) with an 8-0 nylon suture and polyethylene tubing to prevent arterial injury for 30 min, followed by 120 min reperfusion after left lateral thoracotomy. We used electrocardiography to confirm ischemic repolarization changes (ST-segment elevation) during coronary occlusion. Sham rats underwent the same operation, except that the suture was placed around LAD but not tied.

### Histology

To measure myocardial infarction size, hearts were excised and sectioned at 2 mm immediately. Then viable heart slices were stained with 2,3,5-triphenyltetrazolium (TTC), which delineates the infarct region. Images were taken with a Nikon camera and analyzed by using Image-Pro Plus 6.0. To define apoptotic levels of cardiac cells, hearts were extracted and fixed with 4% formaldehyde for 24 h at 4°C before embedding in paraffin for sectioning. Heart tissue was sectioned at 4 μm and underwent staining with an Apoptosis Assay Kit (Roche, Jinan, China). Images were captured by using a microscope (Olympus, X41) and were analyzed by using Image-Pro Plus 6.0.

### Cell Culture

H9C2 cells were cultured in Dulbecco’s modified Eagle’s medium (DMEM) (Gibco, Grand Island, NY, USA) supplemented with 10% (v/v) fetal bovine serum (FBS) and 1% penicillin/streptomycin. All cells were maintained in a humidified incubator with 95% air/5% CO^2^ at 37°C. For *in vitro* study, H9C2 cells were incubated with the ALDH2 activator Alda-1 (20 μM, Sigma, USA) or ALDH2 inhibitor Daidzin (60 μM, Sigma, USA) for 30 min at 37°C (95% air/5% CO^2^) before a 120 min exposure to hypoxia (1% air/5% CO^2^/94% N^2^), followed by 60 min reoxygenation (95% air/5% CO^2^). Furthermore, H9C2 cells were treated with H/R in the absence or presence of Alda-1 with the mitophagy inducer carbonyl cyanide chlorophenylhydrazone (CCCP) (10 μM, Sigma, USA) to evaluate whether and how ALDH2 could affect mitophagy in cardiomyocytes.

### Measurement of ALDH2 Activity

ALDH2 activity was measured in 33 mmol/L sodium pyropho sphate containing 0.8 mmol/L NAD^+^, 15 mmol/L propional dehyde, and 0.1 ml protein extract. Propionaldehyde, the substrate of ALDH2, was oxidized in propionic acid, and NAD^+^ was reduced to NADH to estimate ALDH2 activity. NADH was determined by spectrophotometric absorbance at 340 nm.

### Measurement of Mitochondrial Membrane Potential

H9C2 cells were incubated in the dark and mitochondrial membrane potential was analyzed with an assay kit by incubation with JC-1 (5,5′,6,6′-Tetrachloro-1,1′,3,3′-tetraethyl-imidacarbocyanine iodide, Beyotime, Nanjing, China) in serum-free medium for 20 min at 37°C. Then cells were washed and imaged under a fluorescence microscope (Olympus, X51). Normal mitochondria produce green fluorescence, and depolarized or inactive mitochondria produce red fluorescence. The ratio of red to green fluorescence was analyzed by use of Image-Pro Plus 6.0 and used as an indicator of mitochondrial membrane potential.

### Measurement of Cell Survival

Cell survival was assessed by WST-8 [2-(2-methoxy-4-nitrophenyl)-3-(4-nitrophenyl)-5-(2,4-disulfophenyl)-2H-tetra zolium, monosodium salt] assays using Cell Counting Kit-8 (Solarbio, Beijing, China). H9C2 cells were incubated with 10 μl WST-8 for 2 h at 37°C. The optical density (OD) was measured with a microplate reader (Bio-Rad, Berkeley, CA, USA) at 450 nm.

### Measurement of Reactive Oxygen Species (ROS)

H9C2 cells were incubated with 10 μM dichloro-dihydro-fluorescein diacetate (DCFH-DA) with an ROS assay kit (Beyotime, Nanjing, China) in serum-free medium at 37°C for 30 min in the dark. Then cells were washed and observed under a fluorescence microscope (Olympus, X51) and analyzed by using Image-Pro Plus 6.0.

### Measurement of MitoSOX Red Mitochondrial Superoxide Indicator

H9C2 cells were incubated with 5 μM MitoSOX Red Mitochondrial Superoxide Indicator (YESEN, Shanghai, China) in serum-free medium at 37°C for 10 min in the dark. Then cells were washed and observed under a fluorescence microscope (Olympus, X51) and analyzed by using Image-Pro Plus 6.0.

### Measurement of Mitophagy

The co-localization of mitochondria with mitophagy protein markers was used for detection. H9C2 cells were stained with the mitochondria marker COX IV and mitophagy markers PINK1 and Parkin. The cells were blocked with 5% goat serum and incubated overnight at 4°C with the primary antibody anti-PINK1 (Abcam23707, Rabbit polyclonal to PINK1, Cambridge, MA, USA, 1:100), anti-Parkin (Abcam15954, Rabbit polyclonal to Parkin, Cambridge, MA, USA, 1:200), and anti-COX IV (Abcam33985, Mouse monoclonal to COX IV, Cambridge, MA, USA, 1:100), then with Alexa Fluro 594-Conjugated AffiniPure goat anti-mouse and Alexa Fluro 488-Conjugated AffiniPure goat anti-rabbit secondary antibodies (ZSGB-BIO, Beijing, China, 1:500) for 2 h at room temperature. Then cells were observed under a confocal microscope (LSM710, ZEISS, Germany) at high magnification and analyzed by using Image-Pro Plus 6.0. (The specificity of the antibodies for the protein has not been investigated.)

### Measurement of Apoptosis

Apoptosis Assay Kit (Roche, Jinan, China) was used for TUNEL staining for apotosis. All treated cells were observed under a fluorescence microscope (Olympus, X51) and analyzed by using Image-Pro Plus 6.0.

### Western Blot Analysis

Protein samples were separated by SDS-PAGE and transferred to NC membranes (Millipore), which were blocked with 5% milk and incubated overnight at 4°C with the primary antibody anti-ALDH2 (Abcam108306, Rabbit monoclonal to ALDH2, Cambridge, MA, USA, 1:1000), anti-PINK1 (Cell Signaling6946, Rabbit monoclonal to PINK1, Boston, MA, USA, 1:1000), anti-Parkin (Abcam15954, Rabbit polyclonal to Parkin, Cambridge, MA, USA, 1:1000), anti-4HNE (Abcam46545, Rabbit polyclonal to 4HNE, Cambridge, MA, USA, 1:1000), anti-Actin (loading control, ZSGB-BIO, Beijing, China, 1:1000), and anti-COX IV (mitochondrial loading control, Abcam33985, Mouse monoclonal to COX IV, Cambridge, MA, USA, 1:1000), then with goat anti-mouse and goat anti-rabbit horseradish peroxidase-conjugated secondary antibodies (ZSGB-BIO, Beijing, China, 1:10000) for 2 h at room temperature. After immunoblotting, films were scanned and detected by the chemiluminescence method. (The specificity of the antibodies for the protein has not been investigated.)

### Statistical Analysis

All data are expressed as mean ± SD value of at least three independent experiments. One-way ANOVA was used for comparisons. *Post hoc* Tukey’s test was performed for multiple-range tests. *P* < 0.05 was considered statistically significant. Data were analyzed with use of GraphPad Prism 5 (GraphPad Software Inc., San Diego, CA, USA).

## Results

### Myocardial Infarct Size and Cardiomyocyte Apoptosis in I/R Rats

**FIGURE 1:**
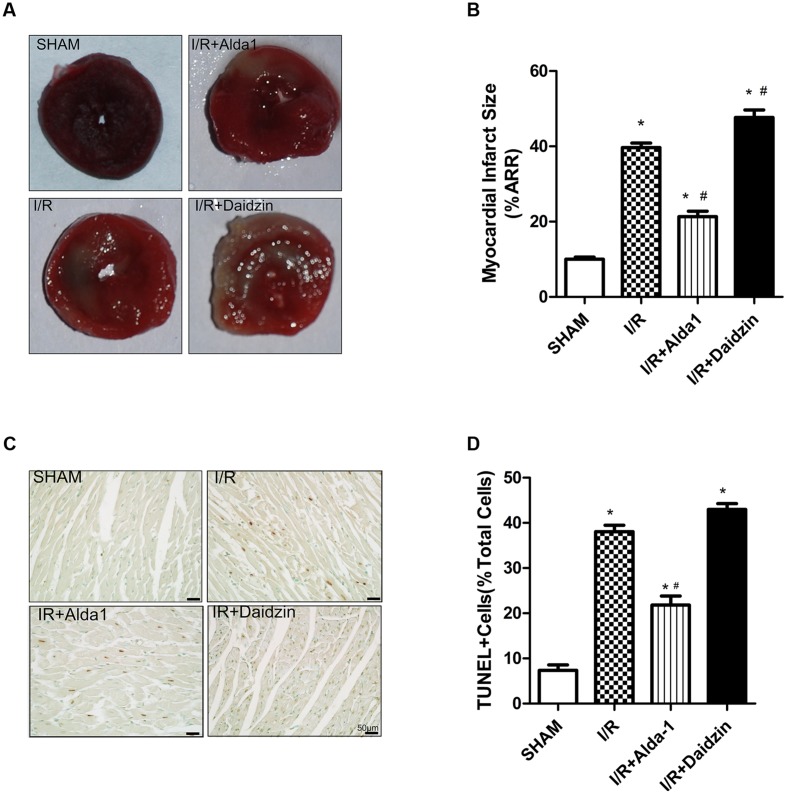
**Myocardial infarct size and cardiomyocytes apoptosis in I/R rats.** **(A)** TTC staining for myocardial infarct size with representative tissue sectioning for SHAM, I/R, I/R + Alda-1, the ALDH2 activator, and Daidzin, the inhibitor. **(B)** The infarct size was expressed as a percentage of area at risk (*P <* 0.0001, One-way ANOVA). **(C)** TUNEL staining for myocardial tissue apoptosis. **(D)** Ratio of apoptosis (*P <* 0.0001, One-way ANOVA). *n* = 3/group. Representative TUNEL staining images are shown (magnification, 200×). Scale bar: 50 μm. Data are mean ± SD from 3 independent experiments. ^∗^*P* < 0.05 vs. sham; ^#^*P* < 0.05 vs. I/R.

We first measured myocardial infarct size in SHAM, I/R, I/R + Alda-1 and I/R + Daidzin groups after 30 min coronary artery ligation and 2 h reperfusion *in vivo*. Compared with I/R alone, I/R + Alda-1 reduced the infarct size and I/R + Daidzin increased the size (**Figures 1A,B**). I/R-induced cardiomyocyte apoptosis was alleviated with I/R + Alda-1. Howerever, it had increasing trend, but no statistical significance with I/R + Daidzin (**Figures 1C,D**). Therefore, ALDH2 plays a key role in myocardial infarct size and cardiomyocyte apoptosis, and ALDH2 activation confers cardioprotection.

### ALDH2 Activity and Expression, 4HNE Accumulation and Mitophagy in I/R Rats

**FIGURE 2:**
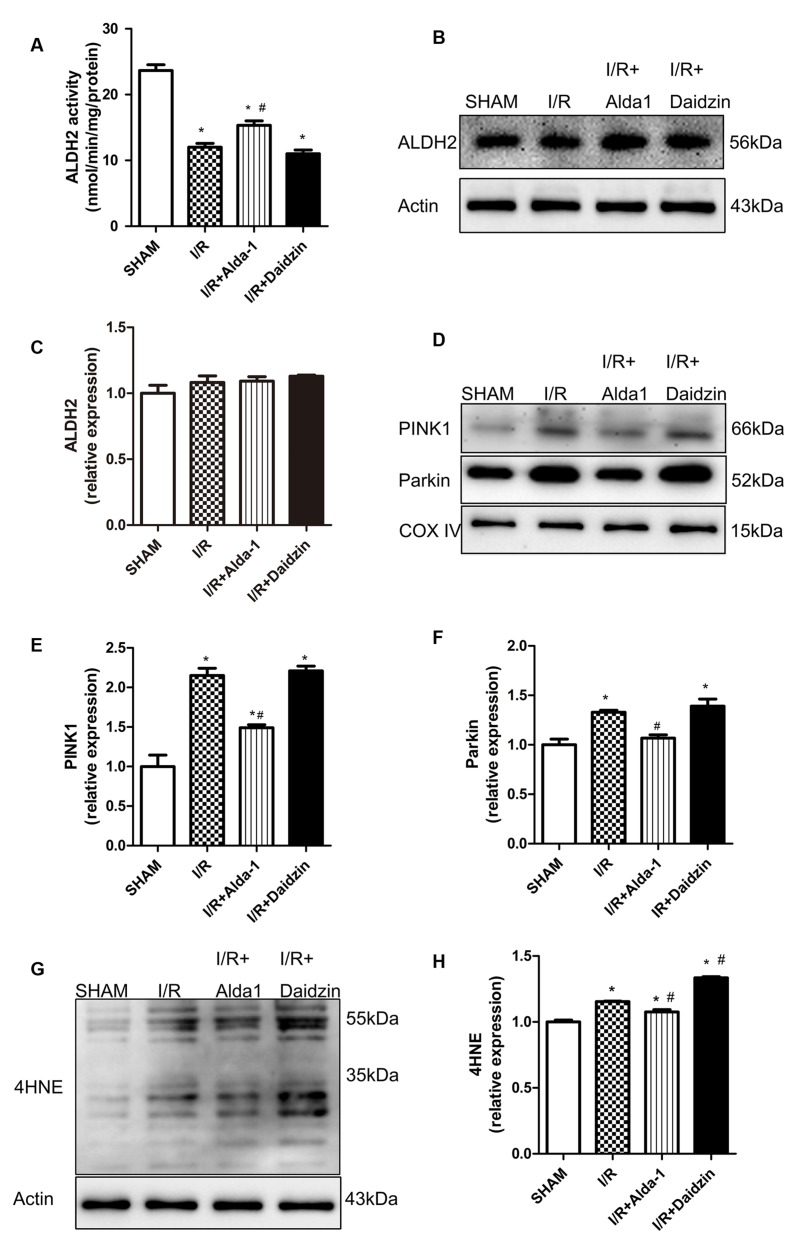
**ALDH2 activity and expression, 4HNE accumulation and mitophagy in I/R rats.** **(A)** ALDH2 enzymatic activity (*P <* 0.0001, One-way ANOVA). **(B)** Representative gel blots of expression of ALDH2 and Actin (loading control). Quantification analysis of **(C)** ALDH2 expression using specific antibodies (*p* = 0.2671, One-way ANOVA). **(D)** Representative gel blots of expression of PINK1, Parkin and COX IV (mitochondrial loading control). Quantification analysis of **(E)** PINK1 expression and **(F)** Parkin expression (*P <* 0.0001, *P* = 0.0012, One-way ANOVA). **(G)** Representative gel blots of expression of 4HNE. Quantification analysis of **(H)** 4HNE expression (*P <* 0.0001, One-way ANOVA). *n* = 3/group. Data are mean ± SD from 3 independent experiments. ^∗^*P* < 0.05 vs. sham; ^#^*P* < 0.05 vs. I/R.

In previous studies, PINK1/Parkin was a critical pathway in controlling mitophagy involved in I/R-induced injury. To explore the potential mechanism(s) under ALDH2-elicited cardiac injury, the lipid peroxidation end product 4HNE, a key ALDH2 substrate, and PINK1/Parkin were evaluated in the rat myocardium. I/R damaged ALDH2 activity, which was significantly alleviated and aggravated trend, respectively, with Alda-1 and Daidzin treatment (**Figure 2A**) with no change in ALDH2 expression (**Figures 2B,C**). To focus on the effects of ALDH2 on mitophagy regulation, we found that I/R increased PINK1 and Parkin levels. ALDH2 activation with Alda-1 suppressed the I/R-elevated expression of PINK1 and Parkin. However, ALDH2 inhibition with Daidzin could not promoted their expression significantly (**Figures 2D–F**). I/R treatment significantly enhanced 4HNE accumulation, which was accentuated and mitigated by ALDH2 inhibition and activation, respectively (**Figures 2G,H**). ALDH2 could play a critical role in mitophagy regulation in myocardial I/R injury and ALDH2 activation protect cardiomyocytes against I/R injury by suppressing PINK1/Parkin–dependent mitophagy via reducing 4HNE accumulation.

### ROS, MitoSOX, Mitochondrial Membrane Potential and Apoptosis in H/R-Treated H9C2 Cells

**FIGURE 3:**
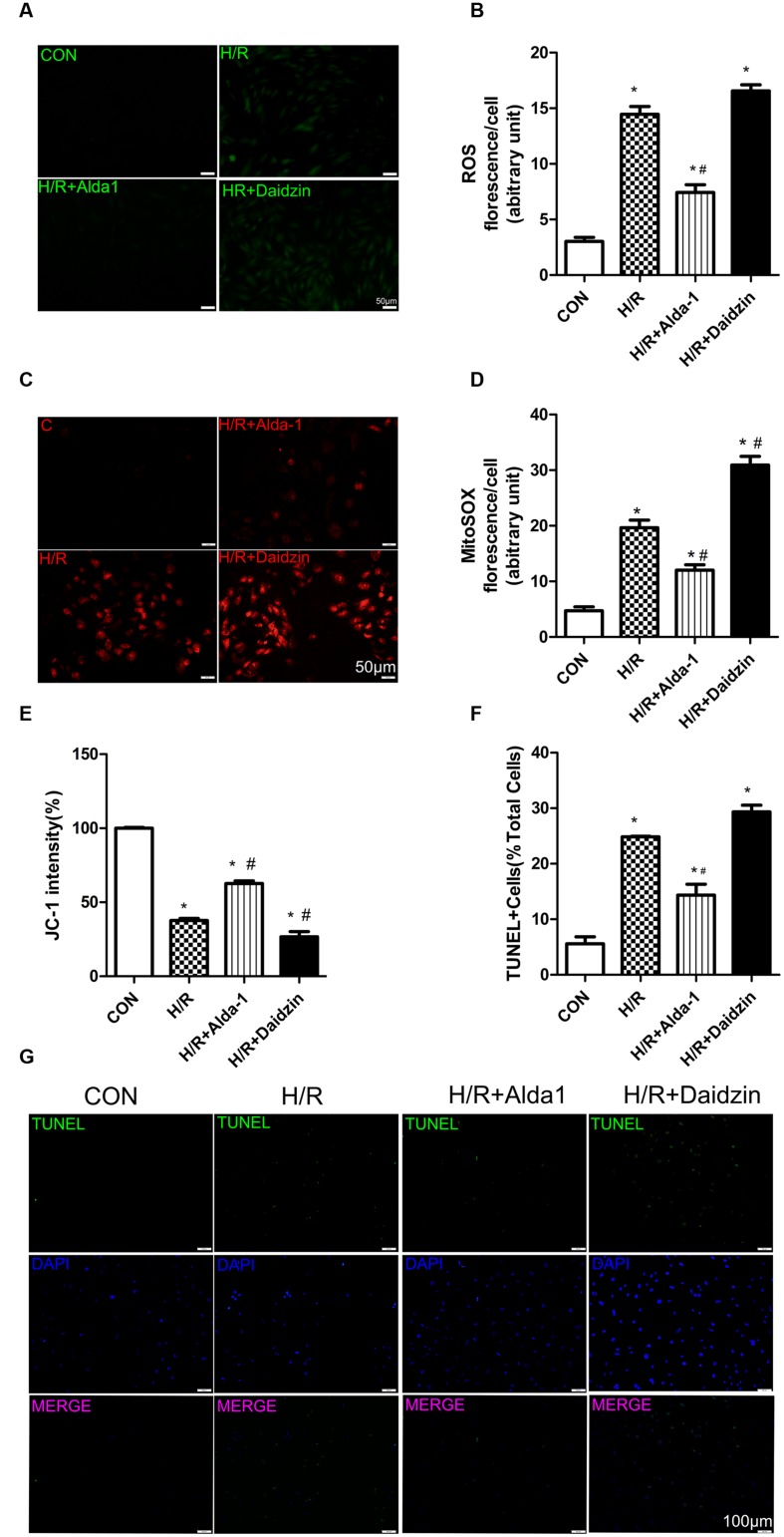
**Reactive oxygen species, MitoSOX, mitochondrial membrane potential and apoptosis in H/R-treated H9C2 cells**. **(A)** DCFH-DA for ROS formation. Representative images are shown (magnification, 200×). Scale bar: 50 μm. **(B)** Quantification of fluorescence intensity of ROS (*P <* 0.0001, One-way ANOVA). **(C)** MitoSOX for mitochondrial superoxide formation. Representative images are shown (magnification, 200×). Scale bar: 50μm. **(D)** Quantification of fluorescence intensity of mitochondrial superoxide (*P <* 0.0001, One-way ANOVA). **(E)** Quantification of JC-1 intensity for mitochondrial membrane potential (*P <* 0.0001, One-way ANOVA). **(F)** Ratio of apoptotic cells to total cells (*P <* 0.0001, One-way ANOVA). **(G)** TUNEL staining for H9C2 cell apoptosis. Representative images are shown (magnification, 100×). Scale bar: 100 μm. Representative fluorescence images and quantitative analysis of 3 independent experiments. Data are mean ± SD from 3 independent experiments. ^∗^*P* < 0.05 vs. CON; ^#^*P* < 0.05 vs. H/R.

Studies have demonstrated that damaged and dysregulated mitochondria generate redundant amounts of ROS, which leads to myocardial damage (Steinberg, 2013). Mitochondria are the major source of intracellular ROS (Starkov, 2008; Kowaltowski et al., 2009). To investigate the relationship between the ALDH2-mediated cardioprotection and oxidative stress, we measured the levels of ROS, mitochondrial superoxide, mitochondrial membrane potential and apoptosis in H/R-treated H9C2 cells. Levels of ROS were higher with H/R than with ALDH2 activation in cells. However, it had the lower trend but no statistical significance with ALDH2 inhibition (**Figures 3A,B**). We also found that I/R increased mitochondrial superoxide levels. ALDH2 activation with Alda-1 suppressed the I/R-elevated mitochondrial superoxide levels but ALDH2 inhibition with Daidzin promoted the levels (**Figures 3C,D**). As well, ALDH2 activation significantly alleviated the decreased mitochondrial membrane potential. (**Figure 3E**). In line with the effects on myocardial I/R injury, ALDH2 activation reduced H/R-induced apoptosis but ALDH2 inhibition could not promoted it significantly (**Figures 3F,G**). Above all, these findings supported a critical role of ALDH2 in H/R-induced injury. ALDH2 activation ameliorated the degree of mitochondrial membrane potential damage and apoptosis by suppressing oxidative stress following H/R challenge.

### ALDH2 Activity, Expression, Cell Viability and 4HNE Accumulation in H/R-Treated H9C2 Cells

**FIGURE 4:**
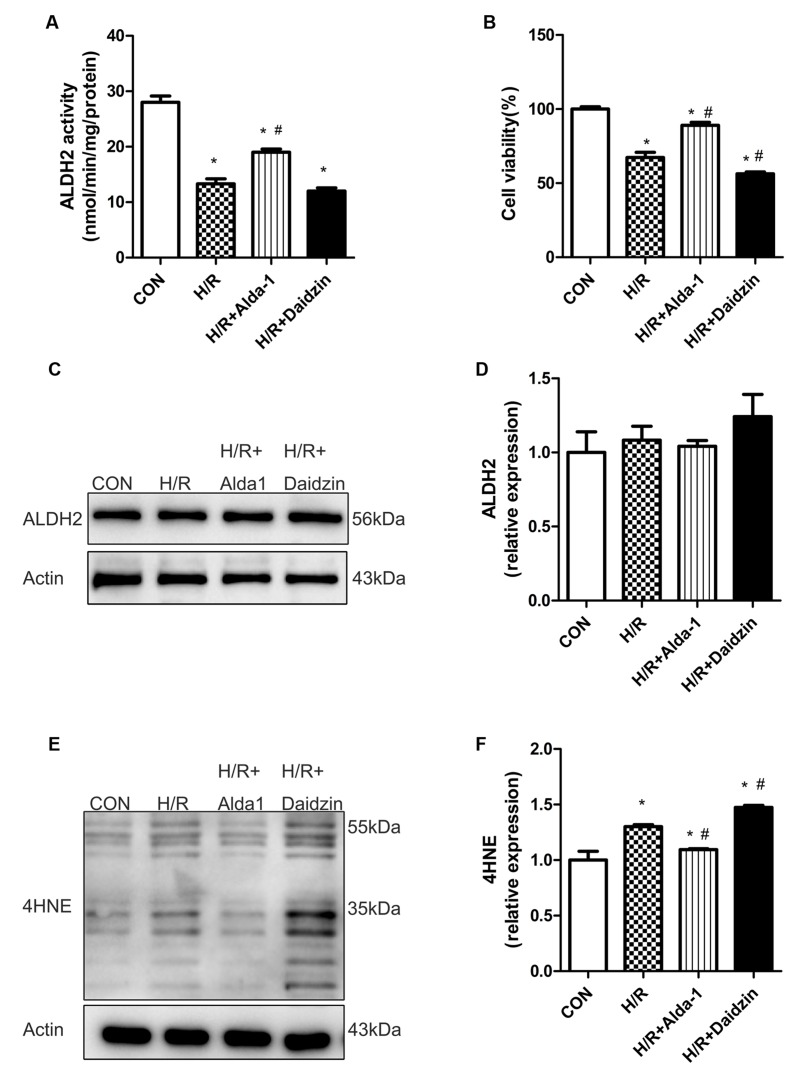
**ALDH2 activity and expression, cell viability and 4HNE accumulation in H/R-treated H9C2 cells.** **(A)** ALDH2 enzymatic activity (*P <* 0.0001, One-way ANOVA). **(B)** Cell viability (*P <* 0.0001, One-way ANOVA). **(C)** Representative gel blots of protein expression of ALDH2 and Actin (loading control). **(D)** Quantification analysis of ALDH2 expression (*p* = 0.5003, One-way ANOVA). **(E)** Representative gel blots of expression of 4HNE. **(F)** Quantification analysis of 4HNE expression (*p* = 0.0002, One-way ANOVA). Data are mean ± SD from 3 independent experiments. ^∗^*P* < 0.05 vs. sham; ^#^*P* < 0.05 vs. H/R.

Consistant with previous results, H/R significantly decreased ALDH2 activity, which was rescued by ALDH2 activation in H/R-treated H9C2 cells (**Figure 4A**) with no change in ALDH2 expression (**Figures 4C,D**). Ratio of cell viability was lower with H/R than with ALDH2 activation in cells (**Figure 4B**). Considering the key role of 4HNE in ischaemic cardiac injury, we examined the effect of ALDH2 on H/R-induced 4HNE protein adduct formation. 4HNE protein adduct formation was significantly increased in response to H/R. Although ALDH2 inhibition significantly promoted H/R-increased 4HNE protein adduct content, Alda-1 further alleviated the increase (**Figures 4E,F**). Thus, ALDH2 activation protected cardiomyocytes against I/R injury by reducing 4HNE accumulation.

### Mitophagy in H/R-Treated H9C2 Cells

**FIGURE 5:**
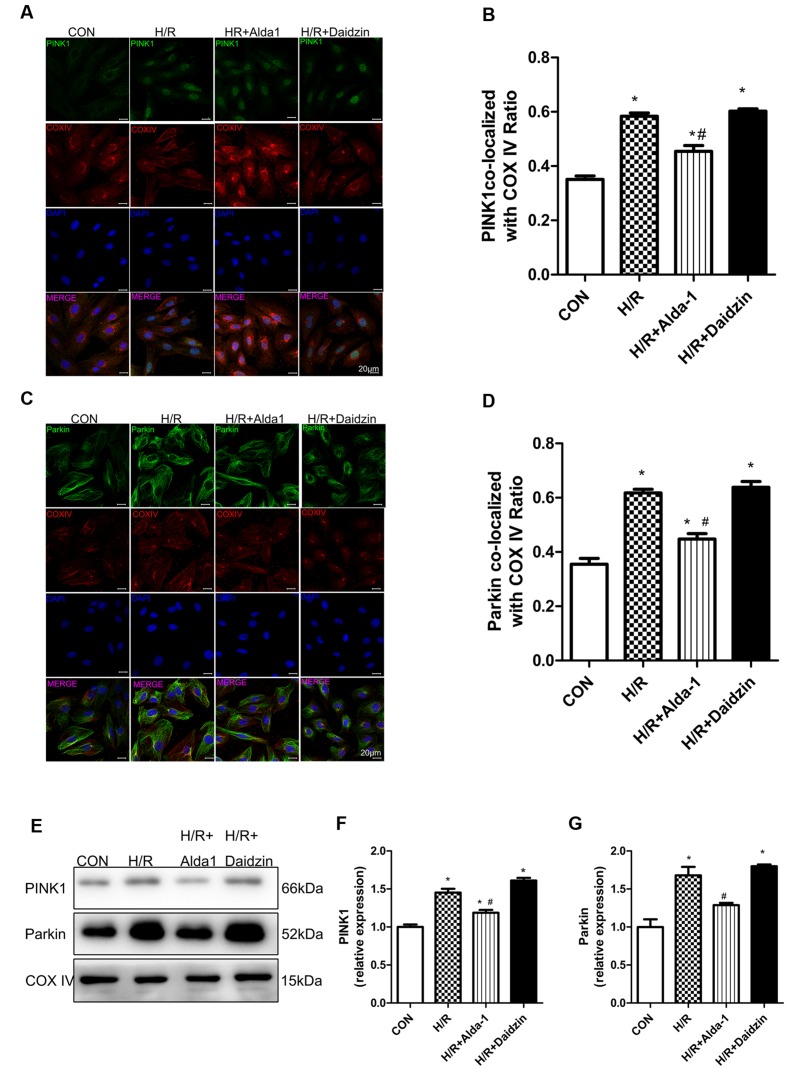
**Mitophagy in H/R-treated H9C2 cells.** **(A)** Representative immunostaining of co-localization of PINK1 and COX IV. **(B)** Co-localization ratio of PINK1 and COX IV. (*P <* 0.0001, One-way ANOVA). **(C)** Representative immunostaining of co-localization of Parkin and COX IV. **(D)** Co-localization ratio of PINK1 and COX IV (*P <* 0.0001, One-way ANOVA). **(E)** Representative gel blots of expression of PINK1, Parkin and COX IV (mitochondrial loading control). Quantification analysis of **(F)** PINK1 expression, **(G)** Parkin expression (*P <* 0.0001, *P* = 0.003, One-way ANOVA). Representative images are shown (magnification, 630×). Scale bar: 20 μm. Representative immunostaining of 3 independent experiments. Data are mean ± SD from 3 independent experiments. ^∗^*P* < 0.05 vs. CON; ^#^*P* < 0.05 vs. H/R.

We then determined the role of PINK1/Parkin in mitophagy induction. H/R increased the co-localization of PINK1 or Parkin with mitochondria, which suggests the activation of mitophagy with H/R; Alda-1 reduced the increased co-localization of PINK1 or Parkin with mitochondria, which suggests suppression of mitophagy activation after H/R (**Figures 5A–D**). As expected, Alda-l reduced this expression induced by H/R. However, Daidzin induced PINK1 and Parkin expression but no significantly (**Figures 5E–G**). Therefore, ALDH2 may protect cardiomyocytes against H/R injury by suppressing the PINK1/Parkin pathway.

### Effect of CCCP on ALDH2-Mediated Cardioprotection in H/R-Treated H9C2 Cells

**FIGURE 6:**
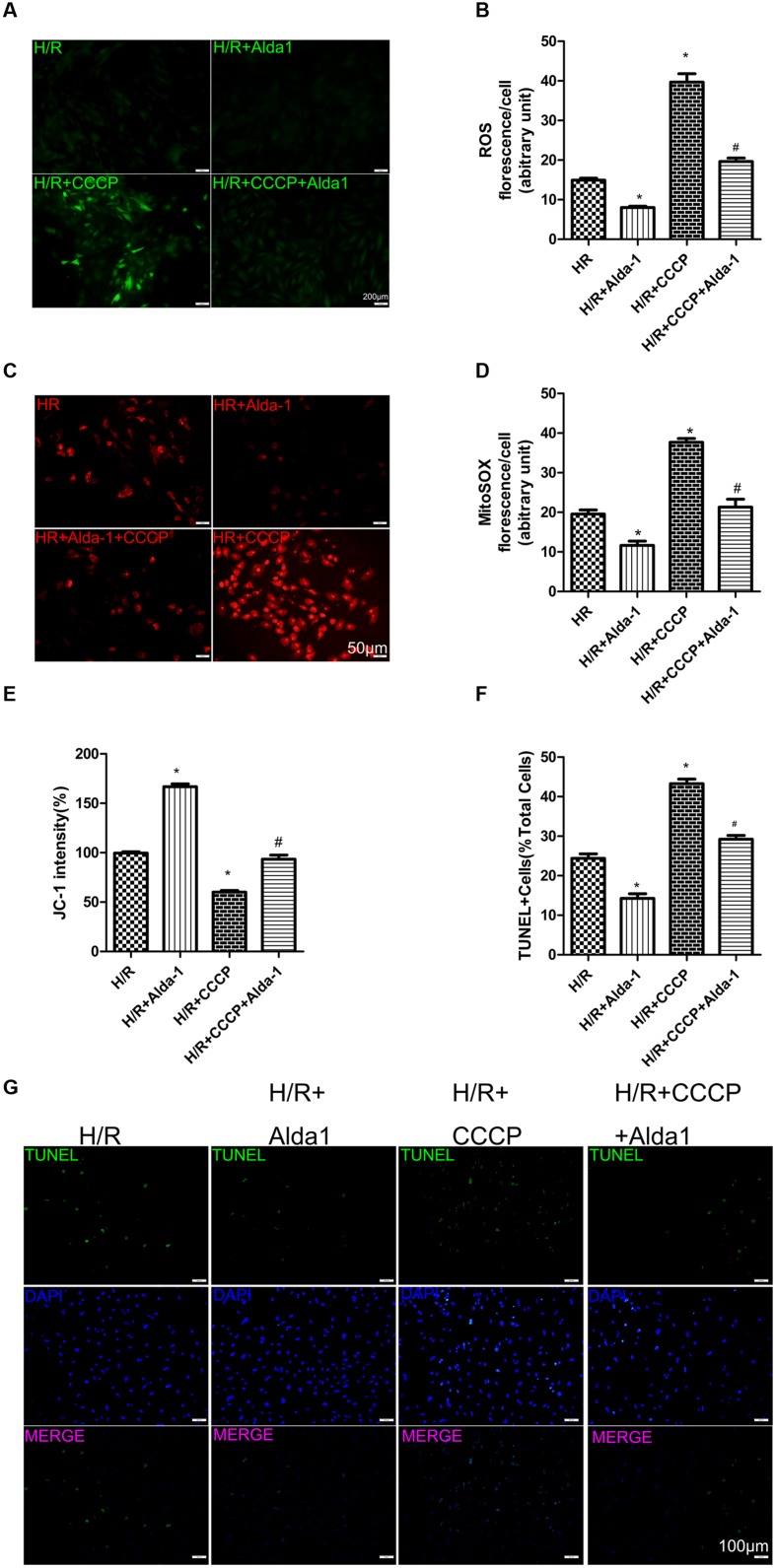
**Effect of CCCP on ROS, MitoSOX, mitochondrial membrane potential and apoptosis in H/R-treated H9C2 cells.** **(A)** DCFH-DA staining for ROS formation. Representative images are shown (magnification, 200×). Scale bar: 50 μm. **(B)** Quantification of fluorescence intensity of ROS (*P <* 0.0001, One-way ANOVA). **(C)** MitoSOX for mitochondrial superoxide formation. Representative images are shown (magnification, 200×). Scale bar: 50 μm. **(D)** Quantification of fluorescence intensity of mitochondrial superoxide (*P <* 0.0001, One-way ANOVA). **(E)** Quantification of JC-1 intensity for mitochondrial membrane potential (*P <* 0.0001, One-way ANOVA). **(F)** Ratio of apoptotic cells to total cells (*P <* 0.0001, One-way ANOVA). **(G)** TUNEL staining for H9C2 cell apoptosis. Representative images are shown (magnification, 100×). Scale bar: 100 μm. Representative fluorescence of 3 independent experiments. Data are mean ± SD from 3 independent experiments. ^∗^*P* < 0.05 vs. H/R; ^#^*P* < 0.05 vs. H/R + CCCP.

**FIGURE 7:**
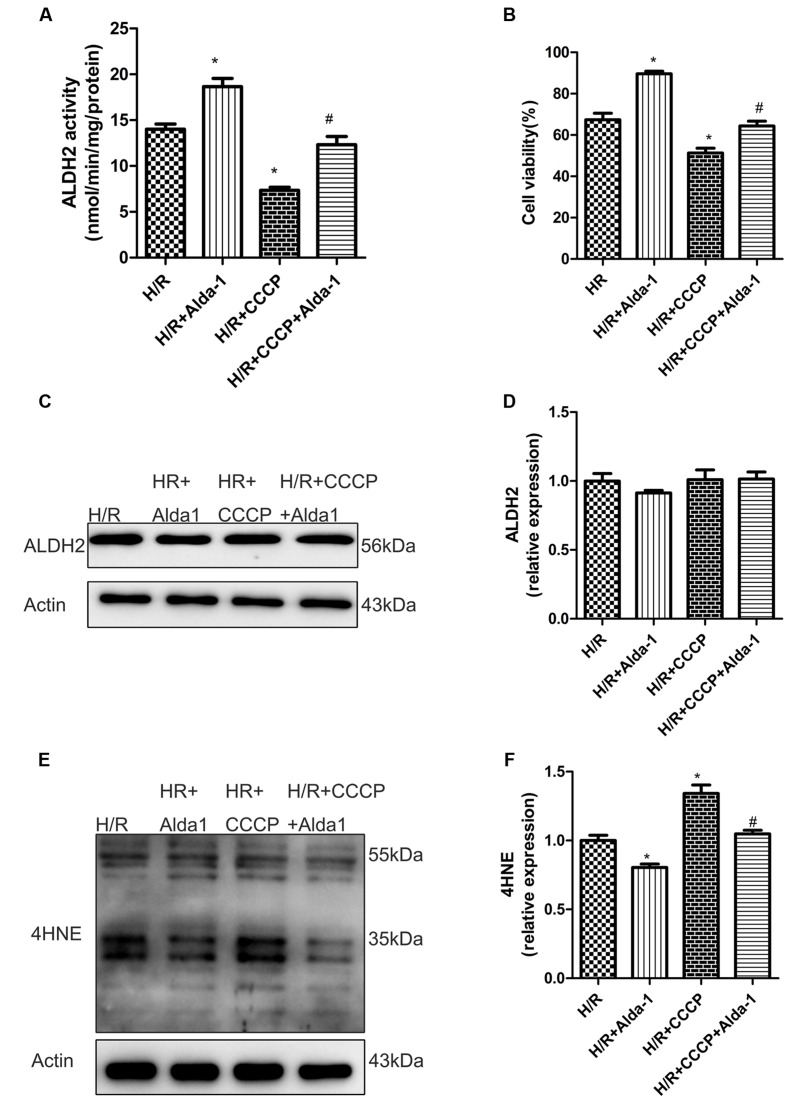
**Effect of CCCP on ALDH2 activity and expression, cell viability and 4HNE accumulation in H/R-treated H9C2 cells.** **(A)** ALDH2 enzymatic activity (*P <* 0.0001, One-way ANOVA). **(B)** Cell viability (*P <* 0.0001, One-way ANOVA). **(C)** Representative gel blots of protein expression of ALDH2 and Actin (loading control). **(D)** Quantification analysis of ALDH2 expression (*P* = 0.5138, One-way ANOVA). **(E)** Representative gel blots of expression of 4HNE. **(F)** Quantification analysis of 4HNE expression (*P* = 0.001, One-way ANOVA). Data are mean ± SD from 3 independent experiments. ^∗^*P* < 0.05 vs. H/R; ^#^*P* < 0.05 vs. H/R + CCCP.

**FIGURE 8:**
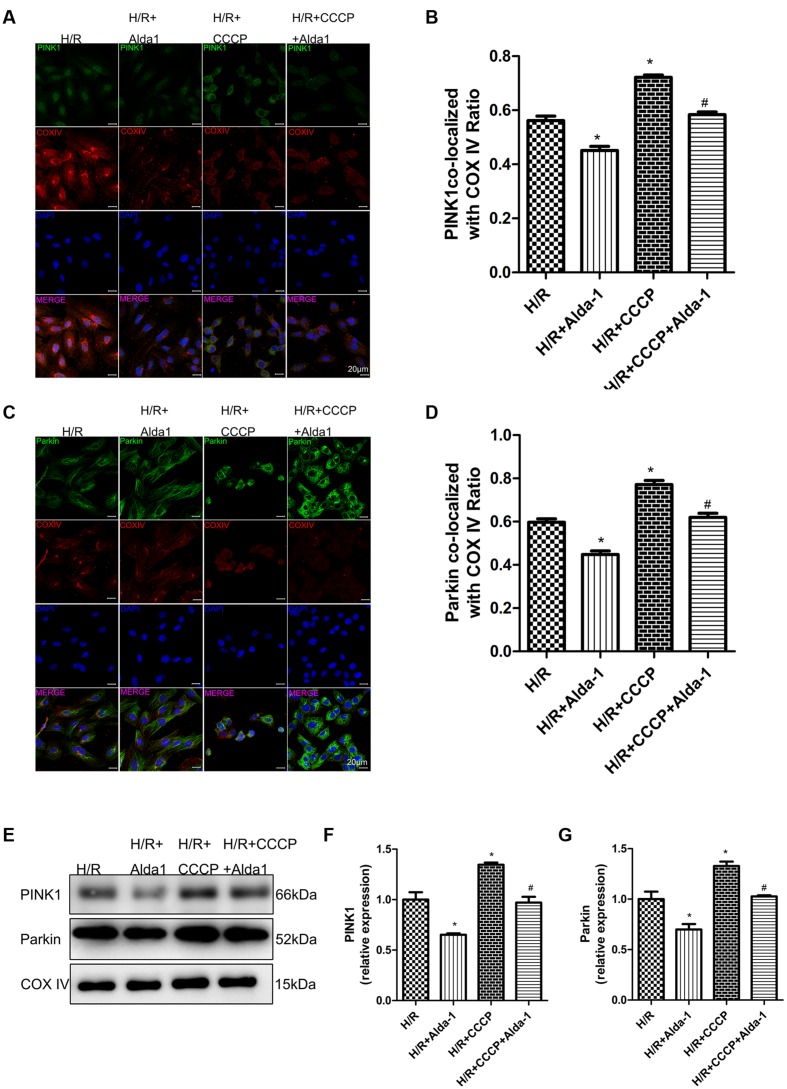
**Effect of CCCP on mitophagy in H/R-treated H9C2 cells.** **(A)** Representative immunostaining of co-localization of PINK1 and COX IV. **(B)** Co-localization ratio of PINK1 and COX IV (*P* < 0.0001, One-way ANOVA). **(C)** Representative immunostaining of co-localization of Parkin and COX IV. **(D)** Co-localization ratio of PINK1 and COX IV (*P* < 0.0001, One-way ANOVA). **(E)** Representative gel blots of expression of PINK1, Parkin and COX IV (mitochondrial loading control). Quantification analysis of **(F)** PINK1 expression, **(G)** Parkin expression (*P <* 0.0001, *P* = 0.002, One-way ANOVA). Representative images are shown (magnification, 630×). Scale bar: 20 μm. Representative immunostaining of 3 independent experiments. Data are mean ± SD from 3 independent experiments. ^∗^*P* < 0.05 vs. CON; ^#^*P* < 0.05 vs. H/R + CCCP.

We used CCCP, an uncoupling protein, to induce mitiophagy in H9C2 cells after H/R. Alda-1 significantly alleviated ROS formation (**Figures 6A,B**), mitochondrial superoxide (**Figures 6C,D**), decreased mitochondrial membrane potential (**Figure 6E**), 4HNE accumulation (**Figures 7E,F**) and apoptosis (**Figures 6F,G**). CCCP accentuated the inhibition of ALDH2 activity (**Figure 7A**), cell viability (**Figure 7B**) after H/R and Alda-1 alleviated the CCCP-induced inhibition with no change in ALDH2 expession (**Figures 7C,D**). Furthermore, CCCP significantly promoted the increased co-localization of PINK1 or Parkin with mitochondria (**Figures 8A–D**) and the expression of PINK1/Parkin after H/R (**Figures 8E–G**) and Alda-1 alleviated the increase. These data indicate that inhibition of mitophagy may be involved in the cardioprotective effect of ALDH2 in H/R injury.

## Discussion

The mitochondrial enzyme ALDH2 has been shown to rescue I/R-induced myocardial injury (Ma et al., 2011). Our present study provided compelling evidence that ALDH2 has protective effects in I/R or H/R-induced myocardial injury by suppressing mitotophagy. The agonist of ALDH2, Alda-1, may offer protection by alleviating I/R or H/R-induced apoptosis via downregulating PINK1/Parkin-dependent mitophagy by eliminating 4HNE accumulation or ROS and MitoSOX formation; the inhibitior of ALDH2, Daidzin, was associated with increased mitophagy activity. Overall, our findings show the pivotal protective role of ALDH2 in myocardial I/R injury by regulating mitophagy.

Many studies have demonstrated that ALDH2 overexpression or activation alleviates myocardial injury induced by I/R (Ma et al., 2011) diabetes, doxorubicin, acetaldehyde, and alcohol (Doser et al., 2009; Ma et al., 2010; Wang et al., 2011; Zhang et al., 2012). Nonetheless, the precise mechanism of the protective effect of ALDH2 on myocardial I/R-induced injury in hearts is unclear. In our study, we found that ALDH2 activation can counteract injury by downregulating mitophagy via eliminating 4HNE accumulation or ROS and MitoSOX formation at both the cardiac and H9C2 cell levels. We also showed the accentuated trend that I/R- or H/R-induced myocardial injury under ALDH2 inhibition. Taken together, our results suggest that ALDH2 plays a pivotal positive role in mediating cardiac protection against ischemic injury.

Mitochondria, as the source of most energy production and endogenous ROS production, play a key role in the functioning and survival of cells (Yan et al., 2013). In general, ROS damage all biomolecules leading to cell death if in overabundance. Mitochondria are the major source of intracellular ROS (Starkov, 2008; Kowaltowski et al., 2009). Some studies demonstrated that leaky type 2 ryanodine receptor (RyR2) cause mitochondrial Ca2^+^ overload, dysmorphology, malfunction and dysfunction in HF (Santulli et al., 2015). They also induce autophagy under pathological conditions of starvation or in myocardial injury after I/R. Moreover, I/R plays a key role in facilitating ROS-dependent lipid peroxidation and production of 4HNE, a highly active carbonyl lipid peroxidation end product (Petersen and Doorn, 2004). 4HNE has cardiac toxicity and impairs ATP production from mitochondria and myocardial contractile function (Aberle et al., 2004). It is one of the most important mediators for lipid peroxidation-derived aldehyde-induced autophagy (Hill et al., 2008). ALDH2 overexpression or activation conferred cardioprotection via detoxification of 4HNE in myocardial injury induced by I/R, doxorubicin, acetaldehyde, alcohol (Doser et al., 2009; Ma et al., 2011; Zhang et al., 2012; Sun et al., 2014). Overexpression or activation of ALDH2 conferred neuroprotection via clearance of 4HNE, whereas ALDH2 knockdown mitigated the neuroprotective property of protein kinase C in the pathogenesis of stroke (Guo et al., 2013). Some studies reported that adduct formation on ALDH itself results in decreased ALDH2 activity and a further rise in 4HNE-induced cell toxicity (Traverso et al., 2002; Aberle et al., 2004). Increased 4HNE adducts on ALDH2 protein in the hearts of mice with symptoms of metabolic syndrome/type-2 DM which may be the reason for the reduced ALDH2 activity (Mali et al., 2014). Alterations of RyR2 and mitochondrial ROS generation form a vicious cycle in the development of AF (Xie et al., 2015). Consistent with previous reports, we observed that H/R induced mitochondrial membrane potential depolarization, ALDH2 activity reduction, ROS production, MitoSOX formation and 4HNE accumulation, which were associated with increased apoptosis in H9C2 cells. Restoration of ALDH2 activity and elimination of ROS and mitochondrial superoxide are therefore critically important.

Mitophagy is an important regulatory mechanism involving selective elimination of dysfunctional mitochondria before activation of cell death in several degenerative diseases (Kubli and Gustafsson, 2012). The PINK1/Parkin pathway is important in regulating mitophagy (Springer and Kahle, 2011). Many studies have shown that the antioxidant superoxide dismutase 2 can squelch mitochondrial targeted photosensitizer-mitochondrial Killer Red (mtKR)–induced PINK1/Parkin-dependent mitophagy, CCCP-induced ROS production increases the Parkin/Pink1-mediated mitophagy in neurons (Wang et al., 2012), which suggests that ROS induced mitochondrial damage may be an important upstream activator of mitophagy. Data from our study show upregulated ROS production, MitoSOX formation, 4HNE level and PINK1/Parkin pathway in I/R-induced myocardial injury. In cultured H9C2 cells, H/R induced significant mitophagy, which was associated with activation of PINK1/Parkin. We also provide direct evidence that ALDH2 activation with the agonist Alda-1 downregulated the PINK1/Parkin pathway and suppressed subsequent apoptosis, ALDH2 inhibition with Daidzin had the trend of upregulateing mitophagy and promoting apoptosis in I/R-induced myocardial injury and HR-induced H9C2 cell injury.

The current experiments involving a mitochondrial depolarizing agent (CCCP) as a mitophagy pathway inducer suggest that the loss of mitochondrial potential can activate mitophagy (Wang et al., 2012). Our CCCP experiments showed that mitophagy was upregulated with CCCP in H/R-induced H9C2 cell injury and ALDH2 activation altered this phenomenon. Thus ALDH2 activation, by suppressing mitophagy, plays a critical role in protecting cardiomyocytes under oxidative stress.

Interestingly, ALDH2 shows dual effects on autophagy in I/R. ALDH2 overexpression moderates upregulated autophagy via activated AMPK to inhibit mammalian target of rapamycin (mTOR) in ischaemia and lessened autophagy via Akt phosphorylation to activate mTOR when AMPK is no longer active in reperfusion phase (Ma et al., 2011). Our data indicate that ALDH2 activation confers cardioprotection through suppressed mitophagy after I/R or H/R. Further studies are required to investigate whether ALDH2 is involved in the paradoxical regulation of mitophagy and how this pathway is regulated by ALDH2 in the ischaemia phase and the reperfusion phase.

Many studies also clearly confirmed the important cardioprotective role of autophagy and mitophagy in the cardiovascular system, and enhancing mitophagy might represent a promising future therapeutic target (Moyzis et al., 2015). Autophagy is increased in the myocardium in response to stress, and this is initially a protective response activated by the cell (Hamacher-Brady et al., 2006). In I/R induced myocardium injury, LC3-II to LC3-I ratio increased in ischemia, decreased in I/R (Ma et al., 2011). Aged Calstabin2 deletion mice impairs autophagy system which displayed increased p62 level, significantly lowered LC3-II to LC3-I ratio, and decreased Beclin-1 level followed by cardiac aging (Yuan et al., 2014). In cerebral-I/R, 3MA and Atg7 knockdown to inhibit autophagy reinforced the brain and cell injury in the reperfusion phase, whereas the mitophagy inhibitor Mdivi-1 in the reperfusion phase and *Park2* knockdown aggravated the ischemia-induced neuronal injury (Zhang et al., 2013). PINK1-deficiency mice increased heart failure more rapidly in response to pressure overload than wild-type mice, and developed the susceptibility of the heart to I/R injury *ex vivo* (Lee et al., 2011; Hoshino et al., 2013). Parkin-deficient mice are more susceptible to doxorubicin-mediated cardiotoxicity (Hoshino et al., 2013). It also ccumulate dysfunctional mitochondria after a myocardial infarction, which leads to increased mortality. In contrast, augmented autophagy and excessive mitophagy is detrimental in a model of pressure overload and reperfusion, which can promote loss of myocytes (Matsui et al., 2007; Lu et al., 2009). However, excessive activation of autophagy induced autophagic cell death in myocardial I/R injury (Matsui et al., 2007; Qiao et al., 2013). Our study indicated that activation of mitophagy was detrimental during I/R and ALDH2 activation alleviated I/R-induced injury *in vivo* and *vitro* by inhibiting mitophagy. Under severe oxidative stress, the PINK1/Parkin pathway may be excessively activated in response to I/R injury to induce myocardial cell death. ALDH2 activation inhibited over-activation of mitotophagy and increased the survival of I/R cardiomyocytes.

More studies are required to fully understand how mitophagy activity and mitochondrial morphology/function is regulated under low and high oxidative stress whether myocardia can be protected by controlling ALDH2 activity and expression. And in our future studies, the antibodies specificity should be investigated using corresponding knockout mice and transgenic cells to consolidate our results.

## Conclusion

In summary, we demenstrate that ALDH2 is cardioprotective in a rat I/R model. ALDH2 inhibited excessive mitophagy and increased the survival of I/R cardiomyocytes by reducing 4HNE and ROS levels. These findings may improve our understanding of the molecular mechanisms responsible for ALDH2 in protecting against myocardial I/R injury and provide new insights for future therapeutic targets. Therapy aimed at potentiating ALDH2 activitaty may be beneficial for in myocardial I/R injury.

## Author Contributions

Conceived and designed the experiments: YC, WJ, and SW. Performed the experiments: WJ. Analyzed the data: WJ. Supervised and guided the experiments: SW, PH, JX, QY, JW, and FX. Contributed reagent/materials/analysis stools: SW, PH, JX, QY, JW, and FX. Wrote the paper: WJ.

## Conflict of Interest Statement

The authors declare that the research was conducted in the absence of any commercial or financial relationships that could be construed as a potential conflict of interest.
